# Yeast gene of unknown function *YGL081W* is involved in DNA damage response

**DOI:** 10.17912/micropub.biology.001647

**Published:** 2025-06-12

**Authors:** Ari King, Taylor M. Emery, Katie Reese, Heather N. Tinsley

**Affiliations:** 1 Department of Biology, Chemistry, Mathematics, and Computer Science, University of Montevallo, Montevallo, Alabama, United States

## Abstract

This study aimed to better characterize gene of unknown function *YGL081W* in *Saccharomyces cerevisiae.* Bioinformatic analysis revealed that *YGL081W* possesses a forkhead associated (FHA) domain, known to be involved in DNA repair and cell cycle arrest. Furthermore, *YGL081W* was predicted to interact with genes involved in cell cycle regulation and its expression changed during mitotic stress. *YGL081W* knockout cells grew modestly better than wild type under normal growth conditions, grew significantly less than wild type when exposed to UV light, and grew no differently than wild type in the presence of hydroxyurea. Collectively, these data suggest *YGL081W* is involved in mediating the DNA damage response in *S. cerevisiae.*

**Figure 1. Variable response of YGL081W knockout cells to mitotic stress:**
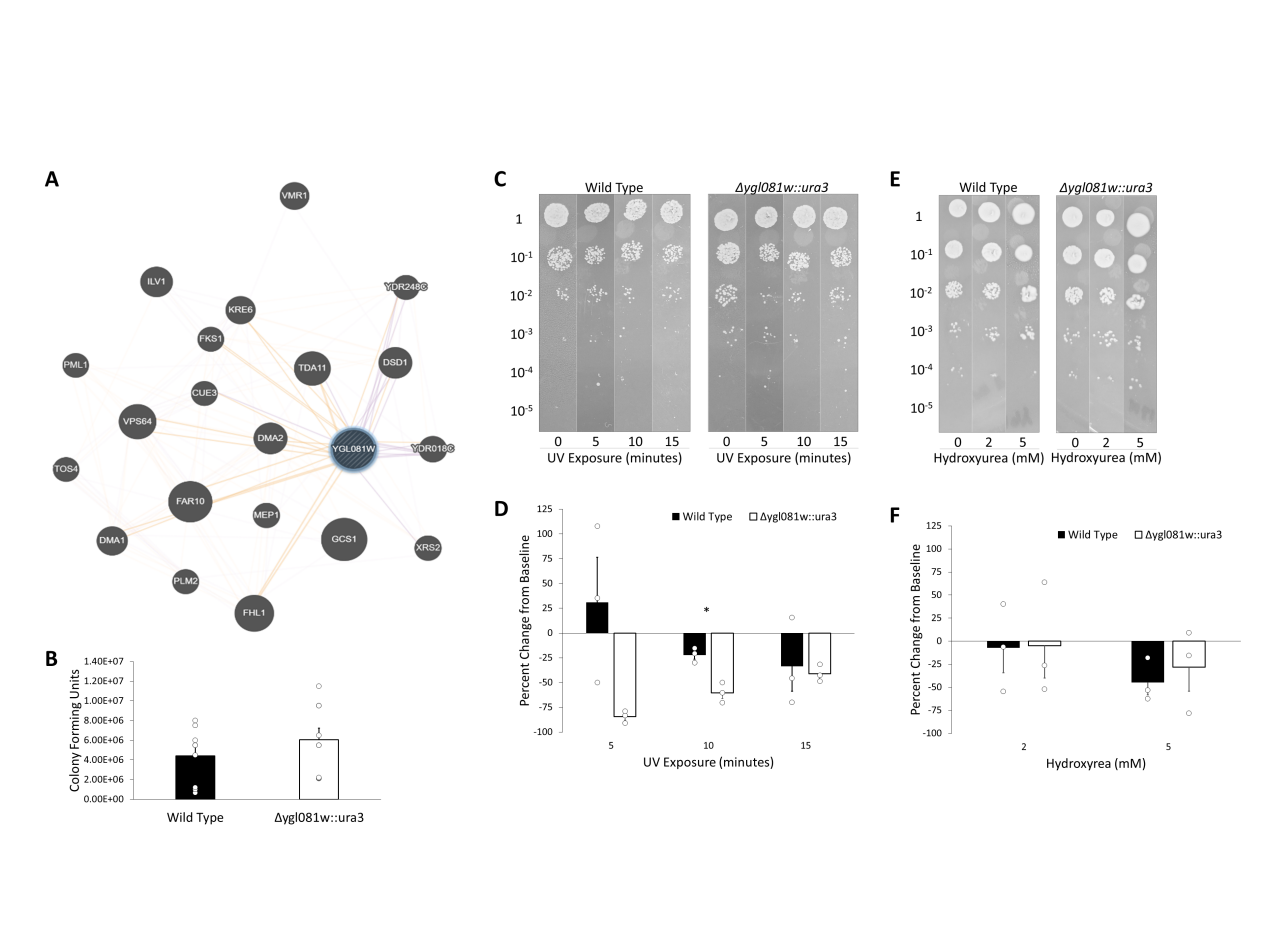
(A) Gene interaction network for *YGL081W* generated by GeneMANIA. Purple indicates genes shown to be co-expressed with *YGL081W* and orange represents genes predicted to interact with *YGL081W* . (B) Bar chart showing number of colony forming units counted for wild type cells compared to *YGL081W::ura3* cells. (C) Representative images from a spot assay performed with wild type and *YGL081W::ura3* cells exposed to 0, 5, 10, or 15 minutes of UV light prior to plating. (D) Bar chart showing the percent change in growth after UV exposure when compared to the unexposed control. Asterisk (*) indicates p<0.003. (E) Representative images from a spot assay performed with wild type and *YGL081W::ura3* cells exposed to 0, 2, or 5 mM hydroxyurea in standard YPD growth media. (F) Bar chart showing the percent change in growth after hydroxyurea exposure when compared to the unexposed control. Bars represent the average of individual points shown. Error bars represent SEM.

## Description

Baker’s yeast, *Saccharomyces cerevisiae* , was the first eukaryotic organism to have its genome fully sequenced (Johnston, 1996). As we approach the 30-year anniversary of this monumental undertaking, nearly 950 of *S. cerevisiae* ’s more than 6,500 open reading frames remain uncharacterized, classified as genes of unknown function (GUFs). Many of these GUFs have been disregarded by the broader scientific community due to their lack of homology in other organisms (Miller et al., 2024). However, yeast such as *S. cerevisiae* are relevant beyond their use as a model organism, playing a foundational role in food and beverage production and biotechnology (Parapouli et al., 2020). As such, understanding the functions of genes unique to *S. cerevisiae* has tremendous scientific and industrial implications.

This study aimed to better characterize one such GUF from *S. cerevisiae* , *YGL081W* . According to the *Saccharomyces* Genome Database (www.yeastgenome.org), *YGL081W* encodes a 320 amino acid, 36 kDa protein with no known homologs. Bioinformatic analysis suggests that this protein is likely expressed in the cytosol with the potential for nuclear translocation. Previous studies showed an interaction with *CHS5* , a gene involved in chitin biosynthesis (Lesage et al., 2005). Additionally, *YGL081W* was found in complex with COP1 during Golgi retrograde transport (Ho et al., 2002). Together, these data suggest a role of *YGL081W* in targeted transport of proteins necessary for cell wall assembly during polarized growth. However, a multitude of studies suggest alternative functions for this GUF. Notably, expression analyses have shown *YGL081W* expression changes in response to a variety of stimuli that are potentially independent of chitin synthesis and cell wall assembly such as aging (Fry et al., 2003), heat shock (Causton et al., 2001; Gasch et al., 2000), and cell cycle arrest (Cho et al., 1998; Spellman et al., 1998; Gasch et al., 2000; Causton et al., 2001).

The protein encoded by *YGL081W* is predicted to possess a forkhead associated (FHA) domain, an 80-100 amino acid, 11-stranded β-sandwich phosphopeptide-binding domain that is known to be involved in signaling related to both DNA repair and cell cycle arrest (Li et al., 2000; Durocher and Jackson 2002). Moreover, as shown in Figure 1A, *YGL081W* is co-expressed with the FHA-containing *XRS2* gene, a necessary component of the Mre11 complex involved in DNA repair (Bressan et al., 1999; Durocher and Jackson 2002; Nicolas et al., 2024). *YGL081W* was predicted to interact with *DMA1* , *DMA2* , and *FAR10* . *DMA1* and *DMA2* are ubiquitin ligases that regulate the mitotic spindle assembly checkpoint (Fraschini et al., 2004; Raspelli et al., 2011; Yoblinski et al., 2021). *FAR10* mediates recovery from cell cycle arrest (Kemp and Sprague 2003). Together, these observations led us to hypothesize that *YGL081W* is involved in stress response during mitosis, specifically in response to DNA damage.

To test our hypothesis, we used site-directed mutagenesis to replace *YGL081W* with *ura3* in the *ura3Δ0* BY4742 strain of *S. cerevisiae* . Once knockout was confirmed using colony PCR, mitotic stress was induced through either DNA damage via UV exposure (Lawrence and Christensen 1976) or inhibition of DNA replication via hydroxyurea treatment (Koç et al., 2004). The impact of knockout on growth in response to these stressors was evaluated via spot assay.

Although not statistically significant, *YGL081W::ura3* cells showed a modest increase in the rate of growth in standard growth media when compared to the wild type BY4742 cells ( Figure 1B ). This suggests that *YGL081W* may possess a growth inhibitory activity under standard growth conditions as removal of the gene resulted in a 37% increase in growth.

Figures 1C and 1D demonstrate the effects of UV exposure on cell growth. Cells were exposed to UV light for 0, 5, 10, or 15 minutes prior to spot assay. As expected, growth of the wild type cells decreased after exposure to UV, and the magnitude of this effect was dependent on the length of exposure. The *YGL081W::ura3* knockout cells showed a more pronounced reduction in growth after UV exposure when compared to the wild type cells, and, interestingly, this effect was the greatest with the shortest exposure times. For example, 10 minutes of UV exposure reduced growth of the wild type cells by 22% and the growth of knockout cells by 60%, whereas 15 minutes of UV exposure reduced growth of the wild type cells by 33% and the growth of knockout cells by 41%. These findings indicate that deletion of *YGL081W* impairs *S. cerevisiae* 's recovery from UV damage, particularly with lower exposure times, suggesting a novel role for this gene in UV damage response.

The effects of hydroxyurea exposure on cell growth were markedly different than that of UV exposure, as shown in Figures 1E and 1F. Cells were exposed to 0, 2, or 5 mM hydroxyurea in their growth media during a spot assay. This exposure caused a dose-dependent reduction in the growth of both wild type and *YGL081W::ura3* knockout cells. There was no statistically significant difference between the effects of hydroxyurea on the *YGL081W::ura3* knockout cells when compared to the wild type cells after correcting for differences in baseline growth. This demonstrates that *YGL081W* is not necessary for mediating cellular responses to the inhibition of DNA replication induced by hydroxyurea.

Cell cycle regulation has been extensively studied in *S. cerevisiae* , but this is the first study to implicate *YGL081W* in the DNA damage checkpoint while also providing evidence that *YGL081W* is not involved in the DNA replication checkpoint. There is overlap in the regulatory mechanisms of individual cell cycle checkpoints, but each of these biochemical pathways is ultimately distinct, allowing cells to respond efficiently and effectively to a variety of insults (Hartwell and Weinert 1989; Nyberg et al., 2002). While we have narrowed down the involvement of *YGL081W* in DNA damage response, the exact mechanism remains unclear.

Interestingly, Rad9, a protein known to interact with the FHA domain of other proteins such as Rad53, demonstrates a very similar effect in the polymorphic fungus *Candida albicans* to what we have shown here with *YGL081W* in *S. cerevisiae. C. albicans* cells with a *RAD9* knockout were unable to perform filamentous growth when exposed to UV, particularly low levels of UV exposure, but remained able to perform filamentous growth when exposed to hydroxyurea (Shi et al., 2007). Like *C. albicans* , *S. cerevisiae* rely on Rad9 for cell cycle regulation in response to DNA damage (Paulovich et al., 1997). Studies are ongoing to determine whether *YGL081W* mediates its DNA damage response through either direct or indirect interactions with *RAD9.*

## Methods

All methods for this project were adapted from the procedures presented as part of the Yeast ORFan Project (Miller et al., 2024).

*Bioinformatics*

Information about *YGL081W* and its encoded protein was obtained from the *Saccharomyces* Genome Database (www.yeastgenome.org). Homology information was obtained by performing a protein-protein BLAST (https://blast.ncbi.nlm.nih.gov/). The BLAST search detected the conserved domain of the FHA superfamily (pfam00498). A gene interaction network was created using GeneMANIA (www.genemania.org). Gene expression analysis was performed using the Serial Pattern of Expression Levels Locator (SPELL) database integrated with the *Saccharomyces* Genome Database (https://spell.yeastgenome.org/). Subcellular localization of the *YGL081W* protein product was predicted using SignalP (https://services.healthtech.dtu.dk/services/SignalP-6.0/), PSORTII (https://psort.hgc.jp/form2.html), NucPred (https://nucpred.bioinfo.se/nucpred/), and DeepLoc (https://services.healthtech.dtu.dk/services/DeepLoc-2.0/).

*Culture Conditions*

*Saccharomyces cerevisiae* strains were cultured in 1% yeast extract, 2% peptone, and 2% dextrose (YPD) with 50 µg/mL ampicillin at 30 ^o^ C. For growth on solid media, 2% agar was added to the YPD media. During knockout, transformed cells were selected on -ura plates, which contained 0.2% Drop-out mixture (US Biological), 0.7% Yeast Nitrogen Base (US Biological), 2% dextrose, and 2% agar with 50 µg/mL ampicillin. Hydroxyurea plates were created by adding filter sterilized hydroxyurea (Thermo Fisher Scientific) suspension to standard YPD agar immediately before pouring plates.

*YGL081W Knockout*

*Ura3-* containing pRS406 plasmid was isolated from *Escherichia coli* cells using the GeneJET Plasmid Miniprep Kit (Thermo Fisher Scientific). Plasmid isolation was confirmed using 1% agarose gel.

For amplification of the *ura3* gene for site directed mutagenesis, a forward primer was designed to recognize the 60 nucleotides immediately upstream of *YGL081W* , which was then combined with the first 20 nucleotides of the *ura3* gene (5’- AAAAAATAATCACTAGGCAGTTGCATAAAGTTGAAACTGAGGGAA GGAACTGGGGAAATCATGTCGAAAGCTACATATAA-3’). A reverse primer was designed to recognize the 60 nucleotides immediately downstream of *YGL081W* preceded by the last 20 nucleotides of the *ura3* gene (5’- TGACTAGCCATGAAGCTATAATACCACCCAGCAATATAGAACTGGGTTATAAAATTATATTTAGTTTTGCTG GCCGCATC). PCR reactions contained 1X PCR Master Mix (Bio-Rad), 0.5 µM of each primer (Eurofins Genomics), and up to 1 ng plasmid DNA. PCR was performed with initial denaturation at 95 ^o^ C for 1 minute followed by 35 cycles of 95 ^o^ C denaturation for 30 seconds, 58 ^o^ C annealing for 30 seconds, and 72 ^o^ C extension for 1 minute, then 72 ^o^ C final extension for 10 minutes. After PCR, samples were incubated with 0.4 units/µL DpnI (New England Biolabs) at 37 ^o^ C for 1 hour to digest remaining plasmid DNA. PCR and DpnI digestion were confirmed using 1% agarose gel.

BY4742 *Saccharomyces cerevisiae* cells were transformed with DpnI-digested PCR reaction mixture using the Frozen-EZ Yeast Transformation Kit (Zymo Research) following manufacturer’s instructions. Briefly, 50 µL of competent cells were transformed with 40 µL of the PCR reaction. A second sample of cells was prepared with water rather than PCR reaction as a negative control. Cells were plated on -ura agar. After 4 days of incubation numerous colonies were apparent on the transformed -ura growth plate while none were apparent on the negative control plate. Five transformed colonies were selected for subculturing.

For confirmation of knockout, a forward primer was designed to recognize the 20 nucleotides upstream of *YGL081W* (5’- GGGAAGGAACTGGGGAAATC-3’). A reverse primer was designed to recognize nucleotides 384-404 of the *ura3* gene (5’- AAACCGCTAACAATACCTGGG-3’). The combination of these primers would produce a 424 bp amplicon only if *YGL081W* was successfully replaced by *ura3* during transformation and recombination. Colony PCR was performed on each of the selected transformants to confirm knockout. Briefly, half of a colony was suspended in 100 µL LiOAc/SDS buffer and incubated at 70 ^o^ C for 5 minutes; 300 µL 95% ethanol was added, samples were centrifuged at 15,000 *g* for 3 minutes, the supernatants removed, the pellets resuspended in 100 µL diH _2_ O, and the samples centrifuged at 15,000 *g* for 30 seconds. PCR reactions contained 1X PCR Master Mix (Bio-Rad), 0.5 µM of each primer (Eurofins Genomics), and 1 µL of the colony lysate supernatant. PCR was performed with initial denaturation at 95 ^o^ C for 1 minute followed by 35 cycles of 95 ^o^ C denaturation for 30 seconds, 53 ^o^ C annealing for 30 seconds, and 72 ^o^ C extension for 1 minute, then 72 ^o^ C final extension for 10 minutes. PCR was confirmed with 1% agarose gel. Appropriately sized amplicons were observed in all samples. One colony was selected and used for all remaining experiments.

*Spot Assay*

A single colony of each wild type and *YGL081W::ura3* cells were selected from YPD plates and cultured overnight in YPD broth. Broth cultures were diluted in YDP broth to an OD _600_ of 0.1. For UV exposure, diluted cells were divided into 60 x 15 mm petri dishes and either immediately prepared for spot assay or placed on a UV light box for 5-, 10-, or 15-minute increments. Serial dilutions were performed at 10X to create dilutions of 10 ^-1^ , 10 ^-2^ , 10 ^-3^ , 10 ^-4^ , and 10 ^-5^ in YPD broth. Undiluted and diluted samples were spotted in 2 µL increments onto YPD agar. For hydroxyurea exposure, diluted cultures were spotted onto YPD agar plates containing 0, 2, or 5 mM hydroxyurea. Each sample for each experiment was spotted in triplicate, and each experiment was performed at least twice. Plates were grown at 30 ^o^ C in the dark for 24-48 hours prior to imaging. Images were analyzed together and representative images selected for inclusion here. Colonies present in the 10 ^-2^ dilution for UV exposure and 10 ^-3^ dilution for hydroxyurea exposure were counted and corrected for dilution factors prior to further data and statistical analysis. Percent change was calculated by subtracting the colony count of the baseline control (no UV exposure or 0 mM hydroxyurea) from the colony count of the replicate matched treatment, dividing by the colony count of the baseline control and multiplying by 100%. Percentages for each replicate were averaged to construct the bar charts. Standard error of the mean (SEM) was calculated in Excel using the sample standard deviation.

*Statistical Analysis*

Differences between wild type and knockout percent change values were evaluated in Excel using a one-tailed, two-sample t-test assuming unequal variances with a hypothesis value of 0.

## Reagents

**Table:** 

**Strain**	**Genotype**	**Source**
BY4742 (Wild Type)	*MATα his3Δ1 leu2Δ0 lys2Δ0 ura3Δ0*	EUROSCARF (www.euroscarf.de)
*YGL081W::ura3*	*MATα his3Δ1 leu2Δ0 lys2Δ0 YGL081W::ura3*	This study

**Table:** 

**Plasmid**	**Description**	**Source**
pRS406	Yeast integrating plasmid with *ura3* selection and β-galactosidase reporter system	ATCC (www.atcc.org)
